# Supplementary figures and images for: Lyophilized powder of calf bone marrow hydrolysate liposomes improved renal anemia: In vitro and in vivo evaluation
Source: PLoS One. 2024 Dec 26;19(12):e0314811. doi: 10.1371/journal.pone.0314811 (PMC11670988; doi:10.1371/journal.pone.0314811)

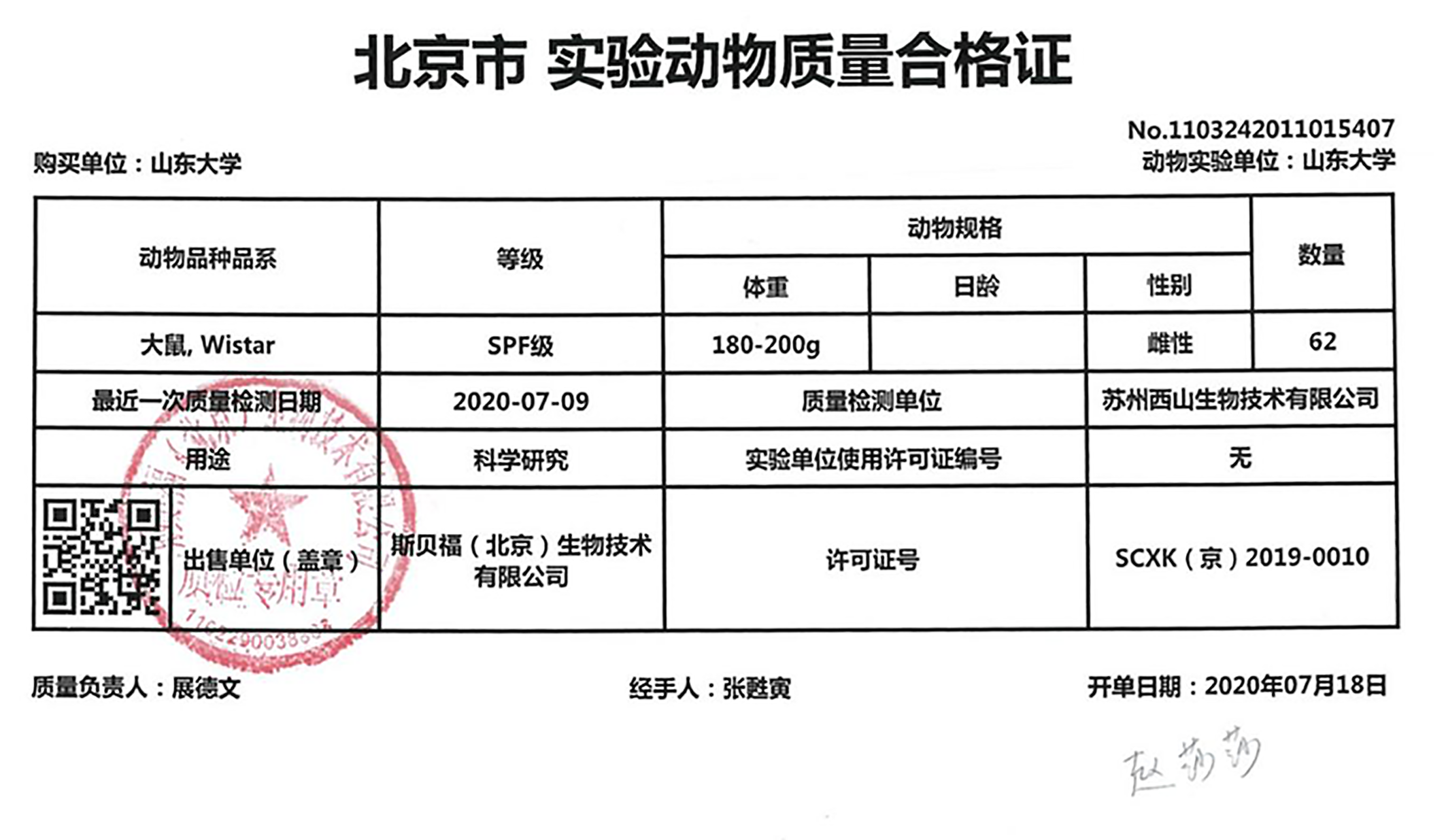

Supplement: S1 Fig — (TIF) [file pone.0314811.s001.tif]

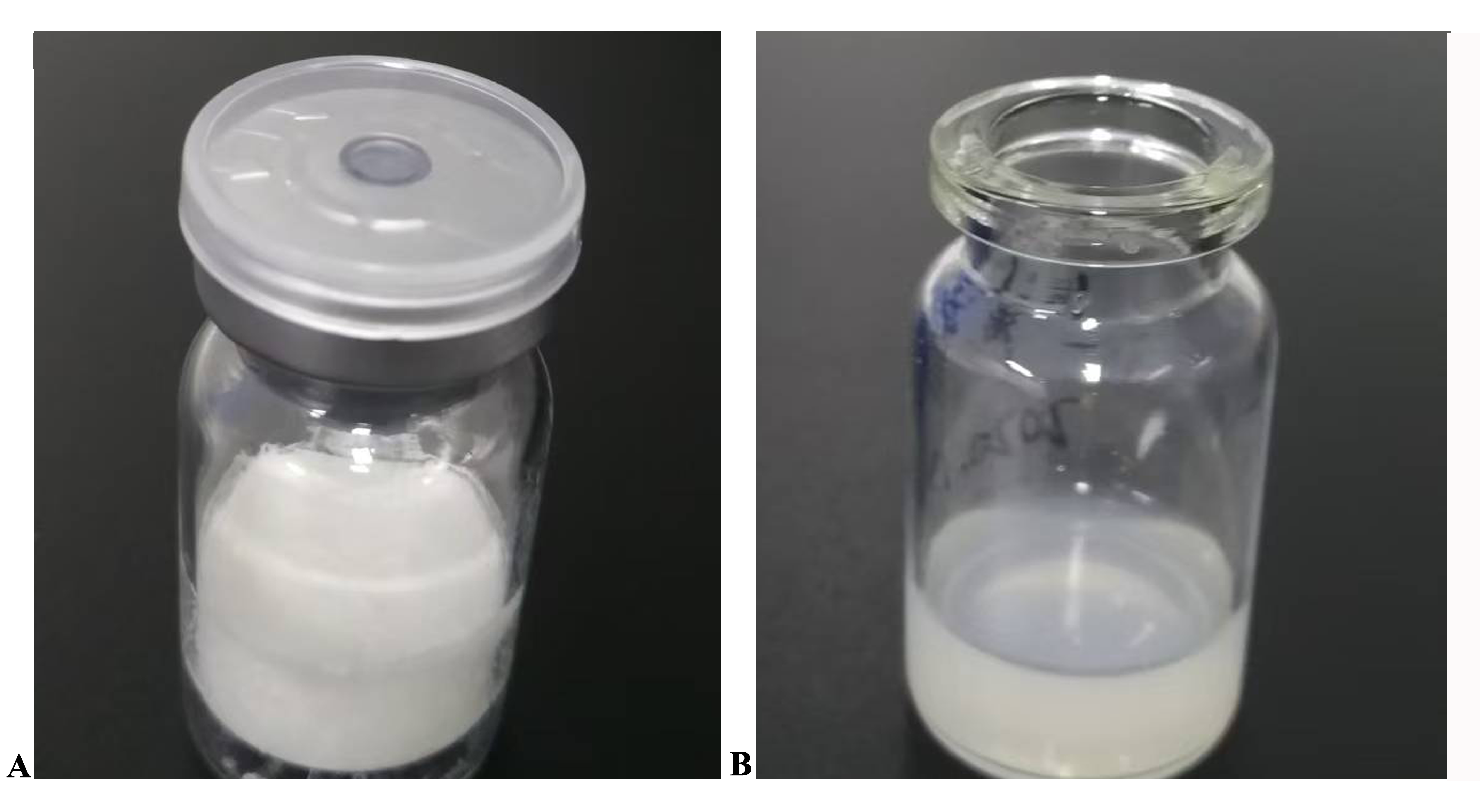

Supplement: S2 Fig — (A) Appearance. (B) Redispersability. (TIF) [file pone.0314811.s002.tif]
