# Supplementary material for: Lyophilized powder of calf bone marrow hydrolysate liposomes improved renal anemia: In vitro and in vivo evaluation
Source: PLoS One. 2024 Dec 26;19(12):e0314811. doi: 10.1371/journal.pone.0314811 (PMC11670988; doi:10.1371/journal.pone.0314811)
Supplement: S1 File — (PDF) [file pone.0314811.s003.pdf]

山东大学齐鲁医院科研伦理委员会

批　准　书

批准号：KYLL-2020(KS)-4018

项目名称： 脐带血造血干细胞治疗大鼠炎性衰老的研究

项目负责人： 李 栋　　**联系电话：** 18560086177

负责研究单位：山东大学齐鲁医院

合作研究单位：

研究起止时间：2020 年 5 月-2025 年 12 月

评审意见：

研究项目：

脐带血造血干细胞治疗大鼠炎性衰老的研究

经伦理委员会审查：

研究者的资格、经验符合研究要求；研究方案符合科学性和伦理原则的要求；同意申报。

山东大学齐鲁医院科研伦理委员会

主任委员：

2020 年 4 月　24 日
