# Supplementary material for: Lyophilized powder of calf bone marrow hydrolysate liposomes improved renal anemia: In vitro and in vivo evaluation
Source: PLoS One. 2024 Dec 26;19(12):e0314811. doi: 10.1371/journal.pone.0314811 (PMC11670988; doi:10.1371/journal.pone.0314811)
Supplement: S2 File — (PDF) [file pone.0314811.s004.pdf]

# 山东大学齐鲁医院实验动物福利伦理审查表

**Application Format for Ethical Approval for Research Involving Animals, Qilu Hospital of Shandong University**

申请日期： 2020 年 3 月 15 日 受理编号：2020-2535 批准文号：DWLL-2020-2535

Appl.Date Y M D Appl.NO. IACUC Issue NO.

| | |
|---|---|
| 课题名称及编号： 小分子多肽治疗肾性贫血大鼠模型的实验研究<br>Program and NO. | |
| 课题来源： 山东省重点研发计划（2019YYSP009）<br>Sponsor | 执行期限：2024 年 12 月<br>Expiration Date |
| 课题负责人：李栋<br>Name of Principal Investigator | 科 室：儿科<br>Department |
| 动物实验负责人：李栋<br>Contact Person | 电话和信箱：lidong73@sdu.edu.cn<br>Contact Tel. No. and Email |
| 课题实施动物实验人数：5<br>Number of Implement | 经专业培训人数：2<br>Number of Certificate |

参与动物实验操作人员姓名、相关专业证书编号，经验、培训、资质和能力的描述：

Name and certificate number, Description of experience/training/competency of the individuals carrying out the research.

李栋：证书编号 20183701210199，熟练掌握各种动物实验操作，指导动物实验进行；

马慧娴：熟悉动物实验方法，有 2 年动物实验操作经历，掌握基本动物操作；

刘玲红：职业兽医师，证书编号 A012018370038，熟练掌握各种动物实验操作，有多年动物实验操作及指导经历；

董娜：熟练动物实验方法，有大于 1 年的动物实验操作经历，能完成基本的动物操作；

朱华甦：熟悉动物实验方法，有大于 1 年动物实验操作经历，能完成基本动物操作。

| | |
|---|---|
| 动物实验设施许可证编号：<br>Name and certificate number of the facility<br>SYXK（鲁）20200022 | 特殊实验设施许可证编号：<br>Name and certificate number of the facility |

现有动物实验设施条件是否与拟开展动物实验的规范性要求相匹配的描述：

Conformity of facility condition and proposed to carry out experiment requirement

齐鲁医院动物实验室具有符合国家标准的实验动物环境设施，实验动物的饲料、垫料、笼器具、饮用水等符合国家标准和相关规定，有健全的实验室管理制度和相应的动物实验操作技能规程。

拟实验时间　　2020 年 4 月 1 日 至 2024 年 12 月 31 日

Experimental period:　　2020 Y　4 M　1 D　to　2024 Y　12 M　31 D

动物实验项目的目的、必要性、意义和如何设计以达成研究目标：

Experimental objective, necessity and significance and how the program has been designed to achieve the objective of the research.

该动物实验拟研究骨髓、胎盘和干细胞来源多种小分子多肽能否缓解腺嘌呤肾性贫血大鼠的多种贫血表现。当前贫血的病因多样化，其中肾性贫血严重危害人类健康。现有常规治疗方案和补血产品只能解决原料短缺问题，但对于造血信号肽相关原料的的补充研究甚少。本研究关注口服小分子造血相关多肽对于促进肾性贫血大鼠康复的疗效，探究其对于红系等多系造血的改善能力。

<table>
<tr><td rowspan="3">拟使用动物信息 Animal to be used</td><td colspan="2">来源：斯贝福（北京）生物技术有限公司<br>Animal origin<br>许可证编号：SCXK（京）2019-0010<br>Certificate number</td><td>质量合格证<br>Certificate of fitness<br>☑有(Yes)<br>☐无(No)</td></tr>
<tr><td colspan="2">品种/品系<br>Breed/strain<br>☑大鼠 rat　☐小鼠 mice<br>☐裸鼠 nude mice　☐兔 rabbit<br>☐犬 dog　☐灵长类 primary animal<br>☐转基因动物 genetically modified animal<br>☐其他（具体说明） others</td><td>等级 Grade<br>☐普通（CV)<br>☐清洁（CL））<br>☑无特定病原（SPF）<br>☐无菌（GF）<br>☐其他（others）：</td></tr>
<tr><td>数量：　36 只（♀）<br>Number　36（♀）</td><td>体重:180-200g<br>Weight 180-200g</td><td>周龄：7 W<br>Age 7 week</td></tr>
</table>

| | |
|---|---|
| | 选择实验动物种类和数量的充分原因：<br>Reasons for the choice of species and numbers of animals to be used.<br>该品系大鼠为高氧损伤模型建立的常用鼠，造模成功率高，模型国际认可度高。 |
| 拟开展动物实验的详细信息（Detailed information of the experiments on animals） | 主要实验操作：Experimental operation<br>1、给药（附件说明：药物名称，确切给药部位，给予体积/次.只，频率，次数等）<br>☑灌胃 ☐皮下注射 ☐腹腔注射 ☐肌内注射 ☐静脉注射 ☐皮内注射<br>☐其他注射途径（请说明）______<br>2、采血（附件说明：采血部位和方法，是否终末采血，是否麻醉，一次/24h累计采血量，频率，次数等）<br>☐耳静脉采血 ☐尾静脉采血 ☐前肢静脉采血 ☐后肢静脉采血 ☑眼底穿刺采血<br>☐其他途径和方法（请说明）______<br>3、采集活体组织（附件说明具体操作）<br>☐尾尖 ☐脚趾 ☐肝脏 ☑骨髓<br>☑其他组织（请说明）肺、心脏、肾<br>4、手术（附件说明：手术名称，主要步骤，麻醉、镇痛药物及方法，术后护理）<br>☑终末性手术（术后不要求动物复苏）<br>☐存活性小手术（不需打开胸腔、腹腔或颅腔，或造成永久肢体损伤，且术后要求动物复苏）<br>☐存活性大手术（需要打开胸腔、腹腔或颅腔，或造成永久肢体损伤，且术后要求动物复苏）<br>5、为辅助实验操作而进行饮食和身体限制（附件说明具体操作和目的及如何监控实施过程）<br>☐禁食 ☐限饲 ☐禁水 ☐限水 ☐器械保定<br>6、行为学项目（附件说明具体操作等）<br>☐水迷宫 ☐高架十字迷宫 ☐穿梭箱 ☐旷场<br>☐其他项目（请说明）______<br>7、心理应激项目（附件说明具体操作及如何监控实施过程等）<br>☐光刺激 ☐声刺激 ☐昼夜颠倒 ☐电击 ☐制动 ☐冷刺激 ☐热刺激 ☐饮食剥夺 ☐社交剥夺 |

| | |
|---|---|
| | ☐其他项目（请说明）__________<br>8、其他操作：请列出操作项目名称，附件逐一说明操作步骤和部位、药物及其用法、实施频率、次数等<br>（没有请填“无”）　无 |
| | 主要观察指标：Main observation target<br>1. 整体生存情况；<br>2. 体重变化；<br>3. 肺、心脏、肾组织病理变化 |
| | 导致疼痛分类：Pain classification:<br>☑轻微，或一过性，或无疼痛<br>☐有疼痛，但能够解除<br>☐不能缓解的疼痛 |
| | 麻醉与镇痛: Anesthesia and analgesia:<br>药物名称：异氟烷　　给药剂量和频率：0-5L/min<br>给药途径：吸入　　维持时间：1h |
| | 仁慈终点或实验终结标准：<br>Humane endpoint or experimental terminative indicator<br>☑ 1. 体重减轻：体重减轻达 20-25%，或是动物出现恶病质或消耗性症候时。<br>☐ 2. 实体瘤的大小超过动物体重的 10%。<br>☑ 3. 丧失食欲：小型啮齿类动物完全丧失食欲达 24 小时或食欲不佳（低于正常量之 50%）达 3 天时。大动物完全丧失食欲达 5 天或食欲不佳（低于正常量之 50%）达 7 天时。<br>☑ 4. 虚弱（无法进食或饮水）：动物在没有麻醉或镇静的状态下，无法进食或饮水，长达 24 小时无法站立或极度勉强才可站立时。<br>☑ 5. 垂死/濒死：动物在没有麻醉或镇静的状态下，表现精神抑郁伴随体温过低（低于 37°C）时。<br>☑ 6. 感染：在抗生素治疗无效并伴随动物全身性不适症状出现时。<br>☐ a.器官：出现器官严重丧失功能的临床症状且治疗无效，或经动物中心兽医师判断预后不佳。 |

☐ b.呼吸系统：呼吸困难、发绀大失血。

☐ c.心血管系统：大失血、已给予一次输液治疗后仍贫血（低于 20%）。

☐ d.消化系统：严重呕吐或下痢，消化道阻塞，套迭，腹膜炎，内脏摘除手术。

☐ e.神经系统：中枢神经抑制、震颤、瘫痪（其中任一肢或以上）、对止痛剂治疗无效之疼痛。

☐ f.肌肉骨骼系统：肌肉受损或骨折使肢体丧失功能。

☐ g.皮肤：无法治愈之伤口、重复性自残或二级以上之保温垫烫伤

☐其他：

---

动物死亡方法：Death conduct

☐ 1. 迅速断头。

☑ 2. 头颈部迅速脱臼（体重＜1kg）。

☐ 3. 二氧化碳或二氧化碳/氧气混合气体。

☐ 4. 在全身麻醉下放血（适合猫、犬和猪等）。

☐ 5. 腹腔注射安乐死药剂。

☐ 6. 静脉注射安乐死药剂。

☑ 7. 过量吸入麻醉剂（氟烷、异氟醚、甲氧氟烷等）。

☐ 8. 其它，具体说明：

---

非处死动物的处置方式：Not for the death of the animal disposition

☐继续使用　　☐◉保存机构

☐放生野外　　☑其他（详细说明）

---

动物尸体、组织、或体液的最终处理

Animal carcasses, organization, or bodily fluids of final disposal

☐ 1. 制作标本。

☑ 2. 袋装后冷冻，由医院统一作无公害化处理。

☐ 3. 其它，具体说明：

---

动物替代、减少动物用量、降低动物痛苦伤害的主要措施：

Major measure for 3Rs

1．实验操作熟练且必要，降低动物的不适感；

2．动物饲养过程中保证饲养条件的规范，防止动物因感染等因素引发的不必要死亡；

3.结合标本取材需要量确定实验组动物数量，不盲目增加组别数

| | |
|---|---|
| | |
| | 是否使用有毒（害）物质（感染、放射、化学、毒、其他）：<br>Poisonous (harmful) material(infection, radiate, chemical poison and other)being used<br>○是 yes ☑否 no<br>说明:<br>Declare |

利害分析的小结，说明为何预期的利益多于害处？

A summary of the herm-benefit analysis-why the expected benefits might be considered to out-weigh the predicted harms?

实验研究建立在大量文献研究的基础上，采用国际认可的高氧损伤新生大鼠模型，通过动物实验证明脐带间充质干细胞治疗高氧所致多器官损伤的疗效，并探究相关机制，实验人员操作熟练，最终实验结果准确性和可获得性得到保障。

信息公开和保密要求:说明哪些信息需要保密，哪些信息可以公开？

Declaration for the information disclosure and confidentiality requirements, declaring the information need to be kept secret, the information can be disclosed

无

其他需要补充说明或辅助证明文件

Supplementary instruction or any auxiliary documents for investigate

无

对伦理委员会有无回避要求：

Claiming jurors for being debarb.

无

| |
|---|
| **声明:1.我将自觉遵守实验动物福利伦理相关法规和各项规定，随时接受伦理委员会和实验动物管理的监督与检查。**<br>**2 本人保证本申请表中所填内容真实，如违反规定，自愿接受处罚。**<br>Declaration: 1. I will abide by the law and regulation stipulation, and accept the supervision and inspection by the committee and laboratory animal department.<br>2. The information I have given is accurate, detailed and comprehensive.<br><br>项目执行人签（章）：<br>Signature(stamp)of Director of project implementation<br>2020 年 3 月 15 日<br>Y M D |
| 实验动物设施意见<br>Opinion from laboratory animal facility<br>设施负责人签(章)：<br>Signature (stamp) of the facility Director<br>2020 年 3 月 20 日<br>Y M D |
| 福利伦理委员会审批意见<br>Approval opinion of Committee<br>通过<br>主任委员签（章）：<br>Signature(stamp)of Chairman of Committee<br>2020 年 3 月 25 日<br>Y M D |
| 备注：☑初审；  □第  次审查。<br>Remarks:☑first trial ;  □reexamine No. |

申报说明：申报时，请提交一式两份及电子版。受理文号和批准文号由伦理委员会填写。

Notice: Submitting the Application Format in duplicate and a electronic edition.
