# Supplementary material for: Lyophilized powder of calf bone marrow hydrolysate liposomes improved renal anemia: In vitro and in vivo evaluation
Source: PLoS One. 2024 Dec 26;19(12):e0314811. doi: 10.1371/journal.pone.0314811 (PMC11670988; doi:10.1371/journal.pone.0314811)
Supplement: Attachment — Submitted filename: Comments.docx [file pone.0314811.s005.docx]

The manuscript titled "***Lyophilized powder of calf bone marrow hydrolysate liposomes improved renal anemia: In vitro and in vivo evaluation***" is suitable for publication in the **PLOS ONE**. However, it must undergo modifications and address both major and minor corrections before it meets the criteria for publication in the mentioned journal.

I suggest approving this manuscript once the following revisions are completed.

**Major comments**

1. In methods section, you didn’t mention any reference(s). Did you perform all methods as first time? You should provide references in this section if necessary.
2. I noticed many grammatical errors through the manuscript. Need to update carefully.
a. Specially the singular and plural form.
b. Active and passive format
c. Mixed with present and past tense in same sentence or on same topics.
3. How you confirmed that the synthesis nanoparticle is liposome not others nanoparticle like LNP? You characterized only by particle size analyzer but didn’t characterize by others analytical tools like TEM or SEM. Additionally you only provide the information for particle size, not PDI, peak intensity (you can refer this article as an example

<https://pubs.acs.org/doi/10.1021/acsabm.2c00061>

<https://doi.org/10.3390/molecules28072969> )

4. You didn’t show any stability data of your formulations. Is there any size differentiation over the time through nano-zetasizer experiments? Is there any size different between drug incorporation and without drugs molecules in liposome? At ease 1 month’s stability data is recommended. (you can use this article as references for formulation stability:

<https://pubs.acs.org/doi/10.1021/acsabm.1c00563>

5. In discussion section, there is lack of scientific and logical explanation based on your research outcome. You mentioned many descriptive information but not sufficient supportive scientific explanations for your research outcome in results section. Please improve the discussion section to support your result.

**Minor comments**

1. In the descriptive sections, you don’t need to mark with identical pools (such as triangle, square, star, or any others marked symbols). In should be as it is that’s are align with previous text or paragraph (line 163-165).
2. How you select dose 250 mg/kg/d? Is there any reference value for this dosage form to get proper pharmacokinetic response? (Line 155)
3. In figure-4, measuring scale is not visualized. Need to make it clear.
4. Funding information is missing.
5. Declaration or conflict of interest is missing.
